# A raça/cor modifica o efeito da mobilidade educacional intergeracional na satisfação com a vida e na renda familiar? Resultados do ELSA-Brasil

**DOI:** 10.1590/0102-311XPT092924

**Published:** 2025-02-24

**Authors:** Bianca Cristina Silva de Assis, Sandhi Maria Barreto, Rosane Harter Griep, Ana Luísa Patrão, Luana Giatti, Lidyane V. Camelo

**Affiliations:** 1 Faculdade de Medicina, Universidade Federal de Minas Gerais, Belo Horizonte, Brasil.; 2 Hospital das Clínicas. Universidade Federal de Minas Gerais, Belo Horizonte, Brasil.; 3 Instituto Oswaldo Cruz, Fundação Oswaldo Cruz, Rio de Janeiro, Brasil.; 4 Faculdade de Psicologia e de Ciências da Educação da Universidade do Porto, Porto, Portugal.

**Keywords:** Grupos Raciais, Racismo, Satisfação com a Vida, Renda, Racial Groups, Racism, Life Satisfaction, Income, Grupos Raciales, Racismo, Satisfacción con la Vida, Renta

## Abstract

Usando dados de 12.987 participantes da segunda visita do ELSA-Brasil (2012-2014), investigamos se a raça/cor (branca, parda, preta) modifica a associação da mobilidade educacional intergeracional com a satisfação com a vida e renda média *per capita*. A mobilidade educacional intergeracional foi avaliada comparando a escolaridade materna com a do próprio participante. Foi utilizada a *Escala de Satisfação com a Vida* (satisfeito *versus* insatisfeito) para avaliar a satisfação com a vida. A prevalência de insatisfação com a vida foi maior em pretos (13,5%) e pardos (12,4%) do que em brancos (9,2%). Após ajustes por idade, sexo, situação conjugal e centro de pesquisa, a mobilidade educacional ascendente foi associada às chances 32% (OR = 0,68; IC95%: 0;53-0;86) menores de insatisfação com a vida quando comparada à trajetória estável-baixa; mas essa associação foi observada apenas entre brancos. A trajetória estável-alta foi associada às chances 31% (OR = 0,69; IC95%: 0,56-0,86) e 29% (OR = 0,71; IC95%: 0,54-0,95) menores de insatisfação com a vida em brancos e pardos, respectivamente. Nenhuma associação entre mobilidade social e satisfação com vida foi observada entre os pretos. De forma consistente com esses achados, as trajetórias educacionais ascendente e estável-alta associaram-se a maior renda familiar *per capita* em brancos e a menor renda em pretos. Nossos resultados reforçam que o racismo estrutural reduz os benefícios da mobilidade educacional em termos de satisfação com a vida e renda em pretos e pardos em comparação aos benefícios observados em brancos.

## Abstract (en)

Using data from 12,987 participants of the second visit of ELSA-Brasil (2012-2014), we investigated whether race/skin color (white, mixed-race, black) modifies the association of intergenerational educational mobility with life satisfaction and average per capita income. Intergenerational educational mobility was assessed by comparing maternal schooling with that of participants. The *Satisfaction with Life Scale* (satisfied versus dissatisfied) was used to assess satisfaction with life. The prevalence of dissatisfaction with life was higher in black (13.5%) and mixed-race people (12.4%) than in white ones (9.2%). After adjusting for age, sex, marital status, and research center, upward educational mobility was associated with 32% odds (OR = 0.68; 95%CI: 0.53-0.86) of lower levels of dissatisfaction with life when compared to the stable-low trajectory, but this association only occurred for white people. The stable-high trajectory was associated with 31% (OR = 0.69; 95%CI: 0.56-0.86) and 29% (OR = 0.71; 95%CI: 0.54-0.95) odds of lower rates of dissatisfaction with life in white and mixed-race people, respectively. Black individuals showed no association between social mobility and life satisfaction. Consistent with these findings, upward and stable-high educational trajectories were associated with higher per capita family income in white people and lower income in black individuals. Results reinforce that structural racism reduces the benefits of educational mobility regarding life satisfaction and income in black and mixed-race people when compared to the benefits for white people.

## Abstract (es)

Utilizando datos de 12.987 participantes de la visita 2 del ELSA-Brasil (2012-2014), investigamos si la raza/color (blanco, pardo, negro) modifica la asociación de la trayectoria educativa intergeneracional con la satisfacción con la vida y el promedio per cápita de ingresos. La trayectoria educativa intergeneracional se evaluó al comparar la educación materna con la de la participante. Se utilizó la *Escala de Satisfacción con la Vida* (satisfecho *versus* insatisfecho) para evaluar la satisfacción con la vida. La prevalencia de insatisfacción con la vida fue mayor entre negros (13,5%) y pardos (12,4%) que en los blancos (9,2%). Después de los ajustes por edad, sexo, estado civil y centro de investigación, la trayectoria educativa ascendente tuvo una probabilidad (32%, OR = 0,68; IC95%: 0,53-0,86) más baja de insatisfacción con la vida en comparación con la trayectoria estable-baja, pero esta asociación se observó solo entre la población blanca. La trayectoria estable-alta tuvo una baja probabilidad del 31% (OR = 0,69; IC95%: 0,56-0,86) y del 29% (OR = 0,71; IC95%: 0,54-0,95) de insatisfacción con la vida entre la población blanca y parda, respectivamente. No se observó una asociación entre la movilidad social y la satisfacción con la vida en la población negra. Estos hallazgos permiten constatar que las trayectorias educativas ascendentes y estables-altas tuvieron una mayor asociación con el ingreso familiar per cápita en la población blanca y un menor ingreso en la población negra. Los resultados refuerzan que el racismo estructural reduce los beneficios de la trayectoria educativa en términos de satisfacción con la vida e ingresos en negros y pardos en comparación con los beneficios observados en la población blanca.

## Introdução

A mobilidade social intergeracional reflete a mudança da posição socioeconômica atual de um indivíduo em relação à posição socioeconômica de seus pais ou responsáveis, indicando a trajetória social de indivíduos e subgrupos populacionais. Geralmente é medida pela comparação da posição socioeconômica do próprio indivíduo, mensurada por indicadores como escolaridade, classe ocupacional ou renda, com a posição socioeconômica de seus pais ou responsáveis. A mobilidade social intergeracional vem sendo associada a diversos desfechos de saúde ^1^ ^,^ ^2^ ^,^ ^3^ ^,^ ^4^ e bem-estar, como a satisfação com a vida ^5^ ^,^ ^6^.

A satisfação com a vida é um componente cognitivo do bem-estar subjetivo frequentemente associado à saúde e longevidade ^5^ ^,^ ^6^. Trata-se de uma medida subjetiva da qualidade de vida que reflete a autoavaliação de domínios importantes da própria vida como trabalho, lazer, cotidiano, entre outros, a partir de parâmetros de referências estabelecidos pela própria pessoa, envolvendo, assim, o julgamento de necessidades, objetivos e desejos alcançados. Por essas características, tende a ser estável ao longo do tempo, sendo por isso usada também como um indicador de desenvolvimento social ^7^ ^,^ ^8^.

O interesse na investigação dos efeitos da mobilidade social na satisfação com a vida tem crescido nos últimos anos, porém os resultados ainda são inconsistentes. Enquanto a mobilidade social descendente tem sido consistentemente associada a menores níveis de satisfação com a vida ^1^ ^,^ ^9^, a direção da associação entre mobilidade social ascendente e satisfação com a vida é incerta. Alguns estudos encontraram que a mobilidade social ascendente foi associada a maior satisfação com a vida ^5^ ^,^ ^10^; entretanto, há autores apontando que os ganhos oriundos da mobilidade ascendente são menores do que as perdas na satisfação com a vida resultantes da mobilidade descendente ^10^. Por outro lado, um estudo que comparou a mobilidade *versus* a imobilidade social, reunindo neste último grupo a imobilidade na alta e na baixa posição socioeconômica, encontrou que a mobilidade ascendente foi associada a maiores chances de insatisfação com vida ^11^. Ausência de associação entre mobilidade social ascendente e a satisfação com a vida também já foi reportada ^12^.

Cabe ressaltar que a variação das categorias de referências utilizadas para a mobilidade social nos diferentes estudos pode explicar uma parcela das inconsistências dos achados, além de dificultar a comparação entre eles ^9^ ^,^ ^11^. Outra explicação para essas inconsistências seria a variabilidade do impacto da mobilidade ascendente de acordo com o contexto social e histórico. Evidências sugerem que ascender na hierarquia social está relacionado a maior acesso a bens, serviços e informações, e a maior potencial para inovação e adaptação de vida ^13^. Por outro lado, ascender socialmente também tem sido associado a sentimentos/percepções negativas e menor bem-estar subjetivo ou apenas este último, já que novas normas sociais podem trazer dificuldade em firmar laços de confiança ^9^ ^,^ ^11^ ^,^ ^12^ ^,^ ^14^.

A investigação da associação entre mobilidade social e satisfação com a vida é predominantemente realizada em países de alta renda, onde as oportunidades de mobilidade social são consideravelmente mais amplas do que em países de baixa e média renda, como o Brasil. Estima-se que, no Brasil, seriam necessárias nove gerações, ou pelo menos 300 anos, para que indivíduos filhos de pais localizados no primeiro decil de renda alcançassem a renda média da população ^15^. Em contrapartida, em países com baixa desigualdade e alta mobilidade, esse período seria de quatro gerações ^15^. Além de ser marcado pelas baixas oportunidades de mobilidade social, o Brasil também é fortemente marcado pelo racismo estrutural que gera, mantém e amplifica as iniquidades raciais ^16^ ^,^ ^17^. Enquanto brancos apresentam maiores chances de ascensão social, pretos e pardos tendem a ser super-representados nas categorias mais baixas da hierarquia social ^18^ ^,^ ^19^. Pretos também experimentam níveis mais altos de estresse relacionados à mobilidade social ascendente do que brancos ^20^, especialmente devido à discriminação racial, que é mais frequentemente reportada entre os pretos de alta posição socioeconômica ^21^. Sabe-se também que os benefícios para a saúde relacionados à mobilidade social ascendente são maiores em brancos do que entre os pretos ^22^. Esse fenômeno é bem descrito e frequentemente referido como “retornos diminuídos relacionados à marginalização” (*marginalization-related diminished returns*) ^23^, já que ele leva a uma associação protetora mais fraca entre indicadores de posição socioeconômica e desfechos de saúde em pretos do que em brancos. Ou seja, o racismo estrutural opera reduzindo as vantagens potenciais da mobilidade social ascendente em pretos quando comparados aos brancos com relação a recursos econômicos e psicológicos ^24^.

Portanto, estudar a associação entre a mobilidade social e a satisfação com a vida em contextos sociais caracterizados pelo racismo estrutural, marcados por desigualdades sociais e menor mobilidade social podem revelar relações mais complexas entre essas variáveis. O objetivo deste estudo foi verificar, em contexto brasileiro, se a raça/cor (branca, parda, preta) modifica a associação da mobilidade educacional intergeracional com a satisfação com a vida e com a renda média *per capita*. Hipotetizamos que, em comparação aos indivíduos com trajetória estável-baixa (permanecer em baixa escolaridade intergerações), as trajetórias sociais ascendentes e estável-alta estão associadas a maiores benefícios em termos de satisfação com a vida e rendimentos médios *per capita* entre brancos, do que entre pretos e pardos.

## Métodos

### População de estudo

Trata-se de um estudo transversal realizado com participantes da segunda visita de exames e entrevistas do *Estudo Longitudinal de Saúde do Adulto* (ELSA-Brasil), realizada entre 2012 e 2014. O ELSA-Brasil é uma coorte multicêntrica de 15.105 servidores públicos, ativos e aposentados, de instituições de ensino e pesquisa de seis capitais brasileiras (Belo Horizonte - Minas Gerais, Porto Alegre - Rio Grande do Sul, Rio de Janeiro, Salvador - Bahia, São Paulo e Vitória - Espírito Santo), com idade entre 38 e 79 anos na segunda visita. Assistentes de pesquisa treinados e certificados realizaram a coleta de dados, que compreendeu entrevistas e exames clínicos e laboratoriais, com rigoroso processo de garantia e controle de qualidade ^25^. Informações adicionais e detalhadas sobre o desenho, método do estudo e perfil da coorte foram descritos em publicações anteriores ^26^ ^,^ ^27^. O estudo foi aprovado pelos comitês de ética em pesquisa (CEP) de cada uma das instituições envolvidas (Universidade de São Paulo - CAAE 0016.1.198.000-06; Universidade Federal de Minas Gerais - Parecer nº ETIC 186/06; Universidade Federal do Rio Grande do Sul - Projeto 06-194; Universidade Federal da Bahia - Registro CEP 027-06/CEP-ISC; Fundação Oswaldo Cruz - Protocolo 343/06; e Universidade Federal do Ceará - Registro CEP-041/06) e todos os participantes assinaram Termo de Consentimento Livre e Esclarecido (TCLE).

Dos 15.105 participantes da linha de base do ELSA-Brasil, 887 não compareceram à segunda visita e 204 faleceram. Dos 14.014 participantes que compareceram à segunda visita, foram excluídos deste estudo aqueles com dados faltantes para satisfação com a vida (n = 85) e raça/cor (n = 157). Foram excluídos também os indivíduos que se autodeclararam amarelos e indígenas (n = 495) por estarem sub-representados na coorte do ELSA-Brasil, dificultando a análise desagregada desses grupos. Além disso, excluímos participantes com dados faltantes de escolaridade materna (n = 290), resultando em uma amostra analítica de 12.987 participantes.

### Variáveis do estudo

#### Variável resposta principal

A satisfação com a vida foi mensurada pela Escala de Satisfação com a Vida (*Satisfaction with Life Scale*) ^7^. Essa escala foi validada previamente em uma grande amostra de adultos brasileiros ^28^. A escala contém cinco afirmativas: “Em geral minha vida está próxima do meu ideal”, “As minhas condições de vida são excelentes”, “Estou satisfeito(a) com a vida”, “Até agora eu consegui as coisas mais importantes que eu quero na vida” e “Se eu pudesse viver minha vida outra vez, eu não mudaria quase nada”. Para cada uma das afirmativas, as opções de reposta variaram numa escala de 1 (discordo totalmente) a 7 (concordo totalmente). Assim, o escore total da escala de satisfação com a vida variou de 5 até 35 - sendo que quanto maior o escore, maior a satisfação com a vida. Neste estudo essa variável foi dicotomizada: satisfeito (escores > 20) e insatisfeito (escores ≤ 20) ^29^ ^,^ ^30^.

#### Variável resposta secundária

A renda média familiar *per capita* foi considerada como variável resposta secundária para auxiliar a compreensão do efeito da raça/cor na relação da mobilidade educacional com a satisfação com a vida. A renda média familiar *per capita* foi estimada pela informação da renda familiar líquida aproximada do mês anterior à entrevista da segunda visita do ELSA-Brasil dividida pelo número de pessoas que dependiam dela. A renda média familiar *per capita* não foi incluída como covariável na análise da variável resposta principal, porque esta sucede a mobilidade educacional, não atendendo assim aos critérios de variável de confusão.

#### Variável de exposição: mobilidade educacional intergeracional

A mobilidade educacional intergeracional foi criada comparando-se a escolaridade materna e a escolaridade atual do participante. A escolaridade materna foi mensurada por meio da pergunta: “Qual é o grau de instrução de sua mãe?” (respostas: nunca frequentou a escola, Ensino Fundamental incompleto, Ensino Fundamental completo, Ensino Médio completo e Ensino Superior completo); e a escolaridade do participante, pela pergunta: “Qual o seu grau de instrução?” (respostas: Ensino Fundamental incompleto, Ensino Fundamental completo, Ensino Médio completo, Ensino Superior completo e pós-graduação).

A escolaridade materna foi categorizada em duas categorias (alta: ≥ Ensino Fundamental completo e baixa: < Ensino Fundamental completo). A escolaridade do participante também foi categorizada em duas categorias (alta: ≥ Ensino Superior completo e baixa: < Ensino Superior completo). Dessa forma, a mobilidade educacional intergeracional foi constituída por quatro trajetórias educacionais: estável-baixa (categoria de referência), ascendente, descendente, estável-alta. A categoria estável-alta refere-se a indivíduos com escolaridade alta tanto para a mãe quanto para o participante. A categoria ascendente descreve indivíduos cuja escolaridade é mais alta que a de suas mães, indicando um progresso educacional. A categoria descendente inclui aqueles cuja escolaridade é mais baixa que a da mãe, indicando regressão educacional. A categoria estável baixa representa a permanência de baixos níveis de escolaridade entre gerações. Essas categorias ajudam a analisar a dinâmica de mobilidade educacional intergeracional.

A categorização da escolaridade materna e da escolaridade do participante utilizou diferentes pontos de corte devido à melhoria contínua dos níveis de escolaridade na sociedade brasileira ao longo do tempo. Em decorrência, a escolaridade média varia substancialmente conforme a coorte de nascimento, sendo muito infrequente a escolaridade média e superior nas coortes mais velhas (maternas). Adicionalmente, sabe-se que o valor relativo das qualificações educacionais diminui à medida que aumenta a escolaridade média na sociedade ^4^.

#### Variável modificadora de efeito: raça/cor

A variável raça/cor autorreferida foi obtida utilizando-se a seguinte pergunta: “O Censo Brasileiro (IBGE) usa os termos ‘preta’, ‘parda’, ‘branca’, ‘amarela’ e ‘indígena’ para classificar a cor ou raça das pessoas. Se o(a) Sr.(a) tivesse que responder ao Censo do IBGE hoje, como se classificaria a respeito de sua cor ou raça?”. A pergunta tinha as seguintes opções de resposta: preta, parda, branca, amarela e indígena. A raça/cor branca foi utilizada como categoria de referência.

### Covariáveis

As covariáveis consideradas para ajuste foram: sexo (utilizado como marcador social de gênero: feminino e masculino), idade, situação conjugal (casado/vive em união, separado/divorciado, solteiro, viúvo e outros) e centro de pesquisa (Minas Gerais, Rio Grande do Sul, Rio de Janeiro, São Paulo, Bahia e Espírito Santo). O gênero, a idade e situação conjugal são variáveis que determinam a mobilidade social além de predizerem níveis de satisfação com vida ^9^. Portanto, são causas comuns da exposição e do desfecho e, por isso, foram consideradas como potenciais confundidoras. Já o centro de pesquisa, consideramos como potencial confundidora por ser um marcador de diferentes oportunidades sociais, como inserções no mercado de trabalho e local de residências, que podem impactar a mobilidade social; e por também refletir contextos culturais distintos, o que poderia influenciar a satisfação com a vida.

### Análise estatística

A análise descritiva da população de estudo foi realizada por meio de proporções para variáveis categóricas e médias e desvio padrão (DP) ou medianas e intervalos interquartis (IIQ) para variáveis contínuas. A prevalência de insatisfação com vida foi descrita segundo raça/cor e mobilidade social.

Para avaliar a associação entre a mobilidade social educacional e o desfecho insatisfação com a vida utilizamos modelos de regressão logística binária com a obtenção de *odds ratios* (OR) e seus respectivos intervalos de 95% de confiança (IC95%). A categoria de referência da mobilidade social educacional em todas as análises foi a trajetória estável-baixa. Após a criação do modelo bruto foi estimado um modelo ajustado pelas covariáveis idade, gênero, raça/cor, situação conjugal e centro de pesquisa. Um termo de interação multiplicativa entre raça/cor e mobilidade educacional foi inserido no modelo final ajustado e encontramos evidências de interação entre raça/cor parda e mobilidade educacional ascendente (p = 0,043) e raça/cor preta e mobilidade estável-baixa (p = 0,009). Por isso, todas as análises foram estratificadas por raça/cor. Não encontramos evidências de interação multiplicativa entre mobilidade social e gênero.

A análise secundária procurou estimar em que medida as diferentes trajetórias de mobilidade educacional dos indivíduos impactaram em seus níveis de renda familiar *per capita*, e verificamos se esse impacto foi diferente entre brancos, pardos e pretos. Inicialmente, descrevemos a mediana da renda familiar *per capita* segundo categorias da mobilidade educacional para cada grupo de raça/cor. Posteriormente, verificamos a associação entre mobilidade educacional intergeracional e o desfecho renda familiar *per capita* (variável contínua) considerando ajustes por sexo, idade, situação conjugal e centro de pesquisa por meio de modelos de regressão linear robusta. Optamos por essa abordagem devido à presença significativa de *outliers* na renda familiar *per capita*, que resultou em grandes resíduos e, consequentemente, em heterocedasticidade. O modelo de regressão robusta foi implementado usando o comando *robreg* no Stata, versão 17.0 (https://www.stata.com), com o método *MM-estimator* e eficiência de 85% ^31^. Esse modelo utiliza uma função de peso que atribui valores menores a observações com resíduos maiores e valores maiores a observações com resíduos menores. Consequentemente, ele reduz o efeito de pontos influentes nos coeficientes de regressão ^32^. Um termo de interação multiplicativa entre raça/cor e mobilidade educacional foi inserido no modelo final ajustado e encontramos evidências de interação entre a trajetória descendente e raça/cor preta (p < 0,001) e parda (p = 0,014) e a trajetória estável-baixa e raça/cor preta (p < 0,001) e parda (p = 0,001). Para facilitar a interpretação dos resultados do modelo final ajustado incluindo o termo de interação, geramos um gráfico com valores preditos de renda familiar *per capita* média para cada uma das trajetórias educacionais em cada grupo de raça/cor utilizando o comando *marginsplot* no Stata 17.0.

Todas as análises foram realizadas no Stata 17.0 e o nível de significância de 5% foi adotado.

## Resultados

Os participantes apresentaram idade média de 55,5 anos (DP = 8,9), a maioria era mulher (54,5%), casado ou vivia em união (66,4%). Mais da metade dos participantes apresentou Ensino Superior completo (54,1%), mas a maioria das mães tinha baixa escolaridade, sendo que 42,8% delas tinha o Ensino Fundamental incompleto e 13,5% nunca frequentou a escola (Tabela 1). A frequência de participantes com Ensino Superior entre os brancos (67,8%) foi mais do que o dobro da frequência observada entre os pretos (28,7%) e situação intermediária foi observada entre os pardos (41,1%). A mesma discrepância verifica-se com relação à escolaridade materna: enquanto 32,1% dos brancos tinham mãe com Ensino Médio completo, apenas 9,6% dos pretos tinham mães com esse nível de escolaridade, e os pardos apresentaram uma frequência intermediária entre brancos e pretos (16,9%) (Tabela 1).

**Tabela 1:** Características descritivas dos participantes, segundo raça/cor. *Estudo Longitudinal de Saúde do Adulto* (ELSA-Brasil), 2012-2014.

Variáveis	Total (N = 12.987)	Raça/Cor		
		Brancos (n = 7.138)	Pardos (n = 3.722)	Pretos (n = 2.127)
Gênero [n (%)]				
Masculino	5.874 (45,2)	46,0	48,2	38,9
Feminino	7.113 (54,8)	54,0	51,8	61,1
Idade [média (DP)]	55,5 (8,9)	56,0 (9,22)	54,6 (8,50)	55,2 (8,55)
Situação conjugal [n (%)]				
Casado/União	8.626 (66,4)	68,2	66,6	60,2
Separado	2.117 (16,3)	15,6	17,3	16,7
Solteiro	1.321 (10,2)	9,9	8,9	13,2
Viúvo	490 (3,8)	3,1	4,1	5,7
Outros	433 (3,3)	3,2	3,0	4,1
Escolaridade participante [n (%)]				
Ensino Fundamental incompleto	623 (4,8)	2,6	7,1	8,0
Ensino Fundamental completo	799 (6,1)	3,9	7,8	10,9
Ensino Médio completo	4.543 (34,9)	25,6	42,9	52,4
Ensino Superior	7.022 (54,1)	67,8	41,1	28,7
Escolaridade da mãe [n (%)]				
Nunca frequentou a escola	1.759 (13,5)	8,0	18,7	22,9
Ensino Fundamental incompleto	5.562 (42,8)	39,1	45,8	50,1
Ensino Fundamental completo	2.539 (19,5)	20,7	18,5	17,4
Ensino Médio completo	3.127 (24,1)	32,1	16,9	9,6
Mobilidade educacional [n (%)]				
Estável-baixa	4.427 (34,1)	22,6	43,7	55,9
Descendente	1.538 (11,8)	9,6	14,2	15,3
Ascendente	2.894 (22,3)	24,6	20,8	17,1
Estável-alta	4.128 (31,7)	43,2	21,3	11,6
Renda familiar *per capita* (R$) [mediana (IIQ)]	2.280 (1.399-3.939)	2.954 (1.865-4.042)	1.865,8 (1.088-2.954)	1.451 (933-2.332)
Insatisfação com a vida [n (%)]	1.413 (10,9)	9,3	12,4	13,5

DP: desvio padrão; IIQ: intervalo interquartil.

Nota: as diferenças nos totais das variáveis ocorrem devido a dados faltantes.

Grande desigualdade racial foi observada na mobilidade educacional, visto que a trajetória estável-baixa e a mobilidade descendente foram muito mais frequentes entre pretos e pardos do que entre brancos. Por outro lado, a trajetória estável-alta e a mobilidade educacional ascendente foram muito mais comuns entre os brancos do que entre pardos e pretos. Ressalta-se que os pardos ocuparam uma posição intermediária entre os brancos e pretos (Tabela 1).

A prevalência de insatisfação com a vida foi 10,9% na população total e apresentou importante variação segundo raça/cor: 9,2% em brancos, 12,4% em pardos e 13,5% em pretos (p < 0,001) (Tabela 1). Diferenças na prevalência de insatisfação com vida segundo categorias de mobilidade social só foram estatisticamente significantes entre brancos, sendo observadas maiores prevalências de insatisfação com vida nas trajetórias descendente e estável-baixa. Entre pretos, essa diferença não foi estatisticamente significante, sendo a prevalência de insatisfação com a vida discretamente maior entre aqueles com trajetória estável-baixa (15,8%) (Tabela 2).

**Tabela 2:** Prevalência de insatisfação com a vida segundo categorias de mobilidade social em brancos, pardos e pretos. *Estudo Longitudinal de Saúde do Adulto* (ELSA-Brasil), 2012-2014.

Trajetórias de mobilidade	Raça/Cor		
	Brancos (n = 7.138)	Pardos (n = 3.722)	Pretos (n = 2.127)
	n (%)	n (%)	n (%)
Estável-baixa	180 (11,2)	198 (12,2)	157 (13,2)
Descendente	97 (14,2)	79 (14,9)	49 (14,9)
Ascendente	136 (7,7)	92 (11,9)	44 (12,1)
Estável-alta	249 (8,1)	93 (11,7)	39 (15,8)
Valor de p *	< 0,001	0,285	0,492

* Teste do qui-quadrado de Pearson.

Os resultados das análises de regressão multivariável, que avaliou a associação entre a mobilidade social educacional e o desfecho insatisfação com a vida, estratificadas por raça/cor mostraram que a mobilidade educacional ascendente em brancos, mas não nos demais grupos raciais, foi associada às chances 32% (OR = 0,68; IC95%: 0,53-0,86) menores de insatisfação com a vida quando comparados àqueles com a trajetória estável-baixa. Já a trajetória estável-alta foi associada às chances 31% (OR = 0,69; IC95%: 0,56-0,86) e 29% (OR = 0,71; IC95%: 0,54-0,95) menores de insatisfação com vida em brancos e pardos, respectivamente. Nenhuma associação entre mobilidade social e insatisfação com vida foi observada entre os pretos (Tabela 3).

**Tabela 3:** Associação entre mobilidade intergeracional educacional e insatisfação com a vida segundo raça/cor da pele. *Estudo Longitudinal de Saúde do Adulto* (ELSA-Brasil), 2012-2014.

Raça/Cor/Mobilidade educacional	Modelo bruto		Modelo ajustado *	
	OR (IC95%)	Valor de p	OR (IC95%)	Valor de p
Brancos				
Estável-baixa	Referência	-	Referência	-
Descendente	1,32 (1,01-1,72)	0,042	1,26 (0,96-1,65)	0,091
Ascendente	0,67 (0,53-0,84)	0,001	0,68 (0,53-0,86)	0,001
Estável-alta	0,70 (0,57-0,85)	0,000	0,69 (0,56-0,86)	0,001
Pardos				
Estável-baixa	Referência	-	Referência	-
Descendente	1,27 (0,96-1,68)	0,097	1,09 (0,81-1,46)	0,520
Ascendente	0,97 (0,75-1,26)	0,830	0,76 (0,57-1,00)	0,040
Estável-alta	0,96 (0,74-1,24)	0,750	0,71 (0,54-0,95)	0,017
Pretos				
Estável-baixa	Referência	-	Referência	-
Descendente	1,16 (0,82-1,64)	0,406	0,98 (0,69-1,40)	0,855
Ascendente	0,90 (0,63-1,29)	0,579	0,77 (0,53-1,11)	0,119
Estável-alta	1,23 (0,84-1,80)	0,282	0,96 (0,64-1,44)	0,808

IC95%: intervalo de 95% de confiança; OR: *odds ratio*.

* Modelo ajustado por gênero, idade, situação conjugal e centro de pesquisa.

**Figura 1:**
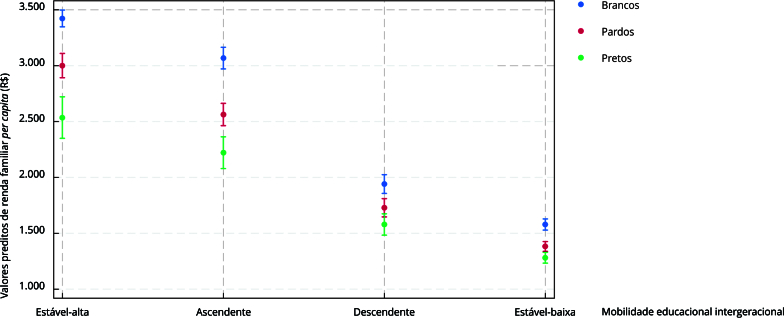
Médias preditas de renda familiar *per capita* segundo mobilidade educacional intergeracional e raça/cor considerando interação raça/cor*mobilidade educacional e ajuste por gênero, idade, situação conjugal e centro de pesquisa. *Estudo Longitudinal de Saúde do Adulto* (ELSA-Brasil).

Constatamos que os maiores níveis de renda familiar *per capita* foram observados entre aqueles com trajetória estável-alta, seguidos pelos indivíduos com trajetória ascendente, descendente e, por último, pela trajetória estável-baixa (Tabela 4). Porém cabe ressaltar que a mediana da renda média *per capita* em todas as categorias de trajetória educacional foi maior para brancos. Os pardos, mais uma vez, assumem uma posição intermediária e os pretos apresentam os retornos financeiros mais baixos em todas as trajetórias. Essas tendências foram corroboradas pelos resultados obtidos pelo modelo de regressão linear robusta ajustado por gênero, idade, situação conjugal e centro de pesquisa ilustrados na Figura 1. Ressalta-se que houve evidências de que a raça/cor preta e parda modificou o efeito da trajetória educacional descendente (preta: p < 0,001; p = 0,014) e estável-baixa (preta: p < 0,001; parda: p = 0,001) o que é ilustrado pelos retornos financeiros mais baixos nessas trajetórias observados nesses dois grupos quando comparados aos brancos.

**Tabela 4:** Mediana da renda familiar *per capita* (R$) e intervalos interquartis (IIQ) segundo categoria de mobilidade social em brancos, pardos e pretos. *Estudo Longitudinal de Saúde do Adulto* (ELSA-Brasil), 2012-2014.

Mobilidade educacional intergeracional	**Renda familiar *per capita* (R$) [mediana (IIQ)]**		
	Brancos (n = 7.138)	Pardos (n = 3.722)	Pretos (n = 2.127)
Estável-baixa	1.554 (1.036-2.280)	1.368 (777-1.865)	1.088 (777-1.865)
Descendente	1.865 (1.119-2.798)	1.554 (1.036-2.280)	1.451 (932-2.176)
Ascendente	3.420 (2.280-5.286)	2.643 (1.710-3.524)	2.280 (1.451-3.109)
Estável-alta	3.524 (2.695-5.908)	2.954 (2.114-3.939)	2.643 (1.617-3.524)

## Discussão

Nossos resultados evidenciam importantes iniquidades raciais na mobilidade educacional intergeracional e na prevalência de insatisfação com a vida, contendo grandes desvantagens para pretos e pardos quando comparados aos brancos. Como esperado, a conversão da escolaridade em benefícios financeiros foi pior para pretos do que para brancos, e os pardos assumiram uma posição intermediária. Da mesma forma, houve menor variação entre as prevalências de insatisfação com a vida segundo as categorias de mobilidade educacional entre pretos e pardos do que para brancos. A análise multivariada confirmou esse achado descritivo ao apontar que os ganhos em escolaridade observados na mobilidade educacional ascendente foram associados a menores chances de insatisfação com a vida apenas entre brancos. Permanecer com alta escolaridade entre gerações (trajetória estável-alta) também foi associado a menores chances de insatisfação com a vida, mas apenas entre brancos e pardos. Nenhuma associação foi encontrada entre as categorias de mobilidade educacional e satisfação com a vida entre pretos. A associação entre mobilidade educacional e a renda familiar *per capita* ajudou a explicar esses achados, já que observamos que as trajetórias favoráveis de mobilidade educacional (mobilidade ascendente e estável-alta) conferem maiores retornos financeiros entre brancos do que entre pretos e pardos, sendo os pretos o grupo em maior desvantagem.

De forma semelhante a outros estudos ^33^ ^,^ ^34^ ^,^ ^35^, encontramos que indivíduos pretos apresentaram maior frequência de trajetórias educacionais desfavoráveis (descendente e estável-baixa) e menores níveis de satisfação com a vida do que brancos e mesmo do que pardos, o oposto sendo observado com relação a trajetórias educacionais favoráveis (ascendente e estável-alta). Esse achado é compatível com estudos representativos da população brasileira que apontam que brancos têm, em média, três vezes mais chances do que pretos e pardos de experimentar a mobilidade educacional ascendente ^36^. Além disso, sabe-se que há uma persistência de alta escolaridade entre gerações nos brancos e que a defasagem escolar é mais frequente entre pretos e pardos ^36^. É importante salientar que a raça/cor da pele antecede a trajetória educacional intergeracional bem como a aferição da satisfação com a vida, podendo, portanto, ser compreendida apenas como um fator confundidor ou modificador do efeito dessa trajetória educacional intergeracional sobre a satisfação com a vida. Neste trabalho demonstramos que a raça/cor da pele modificou o efeito da mobilidade educacional intergeracional na satisfação com a vida.

A educação desempenha um papel importante no sistema de estratificação social e trajetórias educacionais desfavoráveis estão associadas a um menor acesso a recursos econômicos (como bens materiais e simbólicos), recursos psicológicos (como capacidade de enfrentamento e otimismo) e maior exposição ao estresse (como preocupações financeiras e experiências de discriminação) ^5^. Sabemos que a educação formal é o principal caminho para ascender na hierarquia social, mas as pessoas em desvantagens sociais, especialmente as pretas, acabam tendo um desempenho educacional médio menor do que as de maior posição socioeconômica ao longo de toda a vida escolar, de evasão escolar e de serem pais na adolescência, limitando ainda mais suas potencialidades ^37^. Adicionalmente, sabemos que a menor escolaridade pode resultar em um sistema de desvantagem cumulativa, pois está relacionada a piores ocupações, menor renda, moradia em vizinhanças de baixo nível socioeconômico ou algo similar ^3^. Dessa forma, os níveis de escolaridade além de determinarem a posição socioeconômica dos indivíduos, impactam na saúde ao limitar o acesso a recursos que favoreceriam o engajamento em comportamentos protetores ou o controle e a mitigação de consequências de doenças ao possibilitarem acesso aos cuidados em saúde de qualidade ^38^.

Idealmente, seria esperado que o aumento da escolaridade resultasse em melhorias econômicas, sociais, de saúde e bem-estar para todos. Porém, nossos resultados demonstraram que os retornos positivos da escolaridade não foram homogêneos entre os subgrupos de raça/cor, já que as trajetórias educacionais favoráveis geraram retorno em bem-estar em brancos e em menor magnitude entre pardos, e nenhum retorno foi observado entre pretos. De fato, houve uma menor variabilidade nas prevalências de insatisfação com a vida nas trajetórias educacionais avaliadas entre pretos e nesse grupo a maior prevalência de insatisfação com a vida foi justamente na trajetória estável-alta. Em conjunto as menores rendas médias *per capita* e a maior prevalência de insatisfação com a vida observados em pretos, e, em menor magnitude, em pardos, corroboram a teoria dos “retornos diminuídos relacionados à marginalização” segundo a qual o aumento da escolaridade resulta em maior retorno econômico e benefícios para a saúde em brancos do que em pretos ^39^ ^,^ ^40^ ^,^ ^41^.

Os retornos diminuídos observados entre pretos e pardos são em grande parte explicados pelo racismo estrutural, que se refere à totalidade de maneiras pelas quais as sociedades promovem a manutenção de hierarquias raciais e o domínio dos brancos através das gerações ^17^. O racismo estrutural, além de gerar iniquidades de oportunidades, gera desigualdades de resultados, pois mesmo que pretos e pardos alcancem a mesma escolaridade de brancos, terão menores chances de convertê-la em empregos de qualidade, rendimentos, prestígio, entre outros recursos do que os brancos ^42^. Essa pior conversão da escolaridade pode influenciar a satisfação com a vida em pretos e, possivelmente, explica a ausência de associação entre a mobilidade educacional intergeracional e a insatisfação com vida nesse grupo racial.

Cabe destacar também que pretos, mesmo em posições altas na hierarquia social, possuem menos recursos psicológicos e capital simbólico para lidarem com adversidades do trabalho e da vida do que brancos ^43^. Como na categoria de alta posição socioeconômica a frequência de indivíduos pretos é pequena, isso pode resultar em sentimento de “falta de pertencimento”, potencial fonte de estresse e desconforto e um desafio adicional para manter o *status* conquistado ^44^. Além disso, sabe-se que tanto no Brasil ^21^ como nos Estados Unidos, a percepção de discriminação racial é maior entre os indivíduos pretos de alta posição socioeconômica ^45^. Com isso, a manutenção de posições sociais mais altas geralmente implica desafios mais significativos para pretos, o que pode explicar a ausência de diferenças satisfação com a vida relacionada à mobilidade educacional ascendente observada neste estudo.

O único estudo que encontramos que analisou a associação entre mobilidade educacional e satisfação com a vida foi realizado no contexto estadunidense utilizando dados da coorte de idosos *Health and Retirement Study* ^35^. Diferentemente de nossos resultados, nesse estudo os níveis de satisfação com a vida aumentaram à medida que foram observados ganhos em anos de escolaridade dos participantes comparado a escolaridade paterna, tanto em brancos quanto em pretos, apesar dos pretos serem menos satisfeitos com a vida do que brancos. Nessa pesquisa, verificou-se que quanto maior a mobilidade educacional menor era a diferença entre brancos e pretos nos níveis de satisfação com a vida, sugerindo que a mobilidade educacional pode reduzir as iniquidades raciais na satisfação com a vida. Por outro lado, entre os hispânicos, os ganhos em anos de escolaridade dos participantes comparados aos de seus pais foram associados a menores níveis de satisfação com a vida ^35^. Esse achado entre hispânicos é compatível com as conclusões dos estudos de Sorokin ^14^, que apontam que a mobilidade social mesmo ascendente é um evento estressante relacionado à necessidade de adaptação às novas normas sociais que trazem dificuldade em firmar laços de confiança. Isso geraria um estresse social com potencial de precipitar alterações à saúde e afetar o bem-estar subjetivo dos indivíduos ^14^.

Nossos resultados contribuem para uma literatura crescente acerca das iniquidades sociais e raciais na satisfação com a vida. Os pontos fortes deste estudo incluem o tamanho da coorte do ELSA-Brasil, a inclusão de participantes de seis capitais brasileiras, a heterogeneidade racial e a existência de dados detalhados sobre a mobilidade educacional intergeracional. Tais características permitiram examinar e estimar em que medida a mobilidade social intergeracional educacional se relaciona com satisfação com a vida segundo raça/cor. Além disso, cabe destacar que apesar deste estudo ter um delineamento transversal, suas exposições foram fixadas em momento anterior à avaliação da satisfação com a vida e, portanto, a causalidade reversa não seria uma limitação.

A coorte ELSA-Brasil é formada por servidores públicos de instituições de ensino e pesquisa brasileiras com escolaridade média bem superior à encontrada na população brasileira. Com isso, é possível que a prevalência de insatisfação com a vida na população de estudo esteja subestimada em comparação a população brasileira, devido à ausência de indivíduos desempregados e abaixo da linha da pobreza, grupos sociais de grande porte no país. Também não podemos descartar a possibilidade de viés de sobrevida, já que indivíduos de baixa posição socioeconômica, principalmente pretos e pardos, apresentam maior taxa de mortalidade prematura, portanto, têm menores chances de participarem do estudo. Isso poderia levar à subestimação da magnitude das associações observadas.

Em conclusão, nossos resultados mostraram que promover a mobilidade educacional ascendente, apesar de importante, é insuficiente para reduzir a iniquidade racial na satisfação com vida e na distribuição da renda, evidenciando a necessidade de políticas com potencial de reduzir o racismo estrutural e os mecanismos pelos quais ele opera para perpetuar as iniquidades raciais. Dessa forma, os ganhos de escolaridade entre pretos e pardos poderão gerar os mesmos benefícios em satisfação com a vida e em recursos econômicos observados entre os brancos. Portanto, para a promoção da redução das iniquidades raciais na satisfação com a vida e em recursos econômicos é necessário uma transformação e o desmantelamento das políticas e de diversas instituições que sustentam a hierarquia racial no Brasil, já que somente com esse tipo de intervenção será possível modificar valores historicamente construídos na sociedade, que impõe ideias de inferioridade negra e superioridade branca, impactando a vida e a saúde de pretos e pardos entre gerações.
